# Wearable Neuromuscular Electrical Stimulation on Quadriceps Muscle Can Increase Venous Flow

**DOI:** 10.1007/s10439-023-03349-0

**Published:** 2023-08-19

**Authors:** Johanna Flodin, Philip Wallenius, Li Guo, Nils-Krister Persson, Paul Ackermann

**Affiliations:** 1 Integrative Orthopedic Laboratory, Department of Molecular Medicine and Surgery, Karolinska Institutet, Stockholm, Sweden; 2 Department of Trauma, Acute Surgery and Orthopaedics, Karolinska University Hospital, 171 76 Stockholm, Sweden; 3 Polymeric E-textiles Research Group, Swedish School of Textiles, Smart Textiles, University of Borås, Borås, Sweden

**Keywords:** Electrical stimulation therapy, Muscle stimulation, Skeletal muscles, Thromboprophylaxis, Deep vein thrombosis, Venous thromboembolism, Peak venous velocity, Textile electrodes, Smart textiles

## Abstract

Neuromuscular electrical stimulation (NMES) of the quadriceps (Q) may increase venous blood flow to reduce the risk of venous thromboembolism. This study assessed whether Q-NMES pants could increase peak venous velocity (PVV) in the femoral vein using Doppler ultrasound and minimize discomfort. On 15 healthy subjects, Q-NMES using textile electrodes integrated in pants was applied with increasing intensity (mA) until the first visible muscle contraction [measurement level (ML)-I] and with an additional increase of six NMES levels (ML II). Discomfort using a numeric rating scale (NRS, 0–10) and PVV were used to assess different NMES parameters: frequency (1, 36, 66 Hz), ramp-up/-down time (RUD) (0, 1 s), plateau time (1.5, 4, and 6 s), and on:off duty cycle (1:1, 1:2, 1:3, 1:4). Q-NMES pants significantly increased PVV from baseline with 93% at ML I and 173% at ML II. Frequencies 36 Hz and 66 Hz and no RUD resulted in significantly higher PVV at both MLs compared to 1 Hz and 1 s RUD, respectively. Plateau time, and duty cycle did not significantly change PVV. Discomfort was only significantly higher with increasing intensity and frequency. Q-NMES pants produces intensity-dependent 2−3-fold increases of venous blood flow with minimal discomfort. The superior NMES parameters were a frequency of 36 Hz, 0 s RUD, and intensity at ML II. Textile-based NMES wearables are promising for non-episodic venous thromboembolism prevention.

**Supplementary Information:**

The online version contains supplementary material available at 10.1007/s10439-023-03349-0.

## Introduction

Physical inactivity, including immobilization, is a major health threat of the twenty-first century and entails a high risk of venous thromboembolism (VTE) [1, 2]. VTE is estimated to affect one to two individuals per 1000 person-years, with potentially life-threatening consequences [3]. Mechanical prevention of VTE targets venous stasis, the main problem during immobilization, and by increasing venous return using compression of the calf deep vein thromboses (DVTs), the start of VTE can be prevented [4]. Recently, neuromuscular electrical stimulation (NMES) of the calf has been shown as an alternative, mobile technology to improve venous return and potentially prevent VTE [1, 5–8]. Calf-NMES has demonstrated significant increases in the peak venous velocity (PVV) in the popliteal vein, but to lesser extent in the femoral vein [7, 9], where the more dangerous and potentially fatal, DVTs are developed [4]. PVV has been shown to act as a surrogate measure of the VTE-preventive effect [5, 9] and thus means to also increase femoral PVV have been pursued [7, 9, 10].

One study investigating the femoral PVV during simultaneous calf- and quadriceps (Q)-NMES demonstrated a 2.2-fold PVV increase in the popliteal vein, but only a 1.4-fold increase in the femoral vein, compared to baseline [10]. However, whether the PVV increase in the femoral vein was due to compression of the calf or Q-muscle was unclear, since this increase was equal to increases seen in studies examining calf-NMES alone [7, 9, 11]. Thus, to the best of our knowledge, the effect on PVV by Q-NMES alone has not been investigated.

Moreover, PVV increase could be limited due to the known discomfort with NMES [12, 13]. Previous studies have indicated that NMES parameters, such as intensity and frequency, affect the degree of muscle activation and hypothetically blood flow, but also influence the discomfort [14–16]. Additionally, plateau time, duty cycle, and ramp-up/down (RUD) times are speculated to affect discomfort [14, 15, 17]. However, these studies used higher NMES intensities, while recent research indicated that low-intensity (LI)-NMES cause minimal pain [18] and could still significantly increase the PVV during calf-NMES [5–7]. However, the best LI-NMES parameters to increase femoral PVV, while minimizing discomfort are unknown.

Furthermore, compliance to current NMES treatment is low due to difficulties to set up the application in the correct way, including knowledge on where to place the electrodes. In order to simplify NMES-application, facilitate home care and improve compliance [19], we suggest the creation of a wearable easy-to-use, low weight, NMES pants with integrated textile electrodes to enhance femoral blood flow [20, 21].

The primary aim of this study was therefore to assess PVV in the femoral vein during LI-NMES of the quadriceps muscle alone (LI-Q-NMES), compared to baseline, using new NMES pants. The secondary aim was to investigate the best parameters, out of some pre-determined frequencies, RUD times, plateau times, and duty cycles, to maximize blood flow and minimize discomfort. We hypothesized that LI-Q-NMES alone, using the NMES pants, significantly would increase femoral vein blood flow.

## Materials and Methods

### Participants

A total of 16 healthy participants aged between 18 and 60 years were recruited (Table 1). All participants were measured in height and weight and completed a questionnaire about basic characteristic (age, sex, use of tobacco, physical activity level (PAS) [22]).

**Table 1:** Demographics and characteristics of the participants

Variable	Median (IQR) (*n* = 15)
Sex, female, *n*/%	8/53.3
Age, (years)	25 (24–39)
Height, (cm)	173 (169–180)
Weight, (kg)	67 (64–79)
BMI, (kg/cm^2^)	22.6 (21.6–24.8)
Smoker, *n*/%	0/0
Physical activity level*	5 (4–5.5)
Circumference of thigh (cm)^†^	52 (49.5–56)

*BMI* body mass index

*Frändin/Grimby activity scale (1–6)

^†^Circumference of the thigh measures at widest point of each participant’s thigh

An informed consent was signed by all participants before the study confirming that they did not meet any of the exclusion criteria. Exclusion criteria were obesity (body mass index (BMI) > 30 kg/cm^2^), pregnancy, skin ulcer, antithrombotic therapy, vascular abnormalities, previous surgery in the deep vascular system of the leg, pacemaker, intracardiac defibrillator, advanced heart disease, kidney failure, and neuromuscular or metabolic disease. One participant initially recruited was later excluded due to initially failing to recall previous vein surgery to the lower extremity. The sample size was determined based on an estimated twofold increase of PVV from baseline to ML II with the significance level set at *p* < 0.05 and power at 80%, and 11 participants were required to obtain a significant difference. The final sample size was set to 15 participants.

### NMES Pants

A proof-of-concept pair of NMES pants, based on a pair of commercial thigh fitting training shorts, was developed. The pants are made from 83% polyester and 17% elastane in single Jersey, which is a lightweight and thin-knitted fabric construction. Textile electrodes made of commercial conductive fabrics with the trade name Shieldex®fabrics (Statex Productions und Vertriebs GmbH, Bremen, Germany) were designed, manufactured, and integrated into the pants. Electrodes were constructed by sewing to a head-and-tail (external-to-internal) form and using an internal taping to avoid fraying. The square-formed head was designed to ensure good mechanical and electrical contact with the skin, whereas the tail was designed to provide a connector for the NMES source, together forming a single conductive unit.

**Fig. 1:**
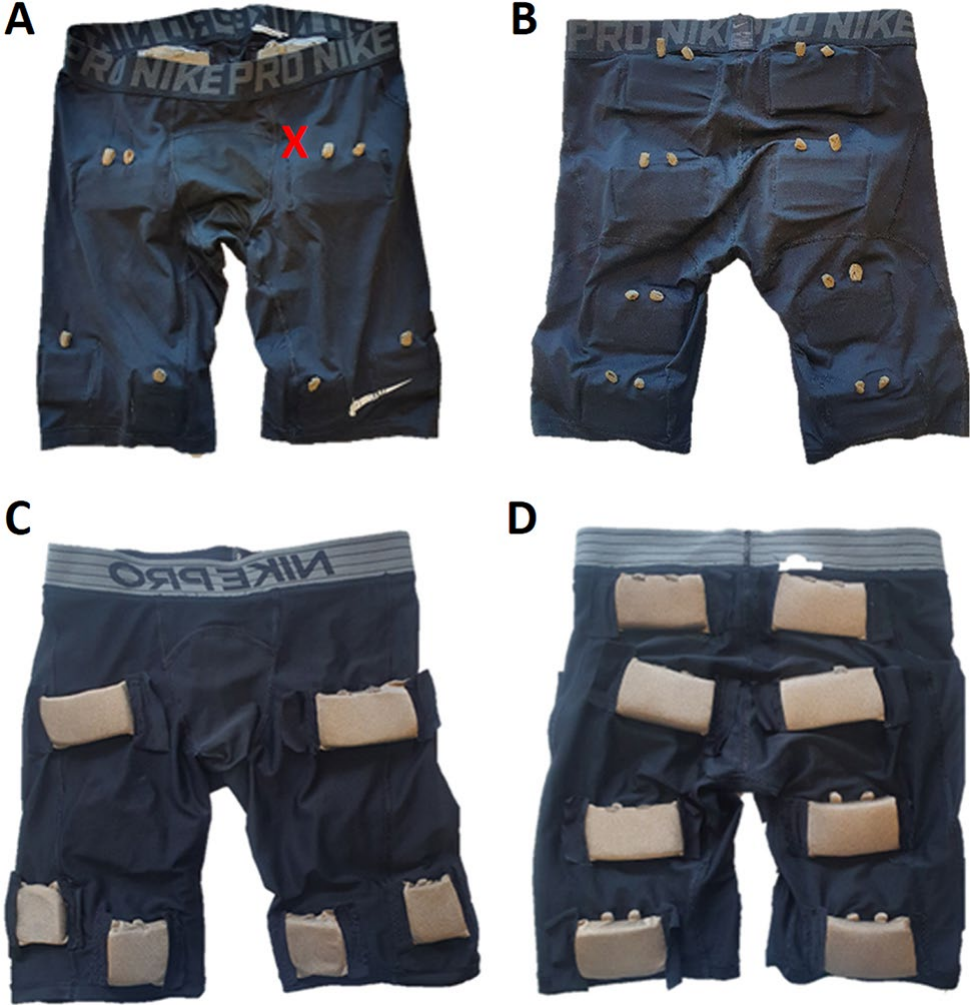
**A**, **B** Pants from the outside (**A** frontside, **B** backside), seen are the connectors for external junctions. **C**, **D** Inside of the pants (**C** frontside, **D** backside). Seen are the side of the electrodes that are applied to the skin, the larger electrodes are sized 5 × 9 cm (upper electrodes in **C** and all electrodes in **D**) and the smaller (lower electrodes in **C**, which were used in this study) are sized 5 × 5 cm. The red “X” in picture A demonstrated the approximate location of the ultrasound probe. The pants are thigh fitting and were adjusted so they had good contact with the skin for all participants

The size and placement of the electrodes in the pants (Fig. 1) were based on a previous study comparing different pre-determined electrode placements and sizes [18]. In this study only the two lower electrodes on the frontside of the pants, sized 5 × 5 cm, covering the distal parts of vastus lateralis and vastus medialis muscles, were used.

### NMES Parameters

**Fig. 2:**
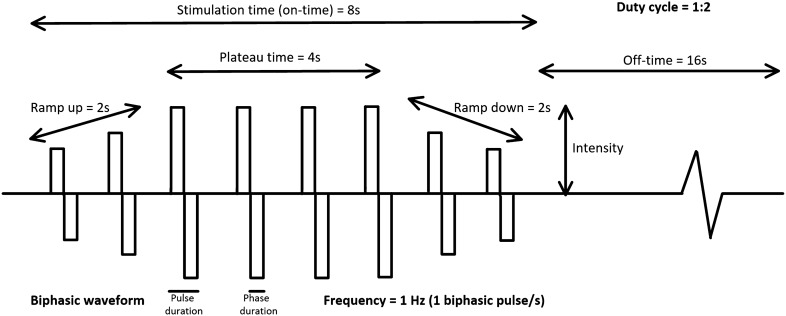
Settings of neuromuscular electrical stimulation (NMES). Schematic illustration of the characteristic of the stimulation during NMES. A biphasic wave pattern is illustrated in the figure, where each impulse includes a positive and negative pulse. The time of each of these are described as phase duration and the time of both the positive and negative pulses as pulse duration. The frequency describes the number of impulses per second. The stimulation time (on time) is the total time for the stimulation, and the ratio between the on- and off-time can be referred to as duty cycle (e.g., 1:3). The stimulation time consists of the plateau time which is the time the stimulation is at the highest stimulation intensity, and ramp-up and down time that represents the gradual increase respective decrease of stimulation intensity

A constant current NMES device, Chattanooga Physio (DJO Nordic, Malmoe, Sweden), using a symmetrical, biphasic, square-form pulse was used. Eleven different combinations of parameters were tested (Table 2). The parameters used to create the eleven tests, frequency (1 Hz, 36 Hz, and 66 Hz), plateau time (1.5 s, 4 s, and 6 s), and ramp-up/down time (0 s and 1 s) (see Fig. 2 for description of the different parameters) were chosen based on those used in previous studies using high-intensity NMES [14, 15, 23–27]. The pulse duration (400 µs) used was the same for all tests. These specific settings were also chosen based on pretesting on several participants, and the different combinations tested was limited since we also noted that prolonged testing time exceeded the endurance of the participants.

**Table 2:** Combination of the NMES parameters tested

Test	Frequency (Hz)	Plateau (on) time (s)	RUD-time (s)	Total on time (s)	Off time (s)	Duty cycle
1	1	2	1	4	8	1:2
2	1	4	0	4	8	1:2
3	36	2	1	4	8	1:2
4	36	4	0	4	8	1:2
5	36	4	0	4	4	1:1
6	36	4	0	4	12	1:3
7	36	4	0	4	16	1:4
8	36	6	0	6	12	1:2
9	36	1.5	0	1.5	3	1:2
10	66	2	1	4	8	1:2
11	66	4	0	4	8	1:2

*RUD-time* ramp-up/ramp-down time, *Hz* hertz

### NMES Measurement Levels

For each test, the NMES level (0–999), representing a non-linear relationship to the intensity ranging from 0 to 120 milliampere (mA), was gradually increased by two NMES levels at a time, until measurement level I (ML I) and measurement level II (ML II) was reached, at which point the data for the outcomes were registered (PVV, discomfort and intensity). ML I was defined as the minimum NMES level required to induce a visible coherent contraction of the whole muscle observed by the same trained investigator for all participants [18]. The NMES level was then increased six additional steps on the NMES device from ML I, and this level was defined as ML II. These two MLs were chosen based on previous studies investigating hemodynamics during calf-NMES that demonstrated that similar intensities produced a significant increase in PVV [5, 6]. In a previous study on the quadriceps, the intensities representing ML I and ML II have been shown to produce minimal discomfort [18]. The NMES levels were then translated into mA based on information from DJO Global.

Due to the non-linear relationship between the NMES level and the intensity, and the fact that different participants required different intensities to reach ML I median (min-max) 14 (9.9–22.4) mA and ML II 19.5 (15.8–26.3) mA, the constant 6 NMES level increase between ML I and ML II represented different increases of intensity for different participants. The increase of intensity (mA) was 5.5 (3.8–6.5).

### Assessment of Hemodynamics

A Philips CX50 (2013) Doppler ultrasound machine (Philips Medical Systems, Andover, MA, USA) was used for the hemodynamics measurements in the femoral vein. The ultrasound measurements were performed through the thin NMES pants, using ultrasound gel applied on the NMES pants. Prior to the study, we performed pretesting with and without pants, which demonstrated equal results in the ultrasound Doppler measurement (Supplementary Table 1) and also consulted a professor in radiology to ensure correct measurements. The measurements of PVV were assessed at the widest accessible part of the femoral vein at the proximal part, approximately 5 cm down from the inguinal fold and visualized in a longitudinal plane with the ultrasound with the same procedure as described in the previous studies [28, 29]. Recordings of blood flow in cm/s were saved on the ultrasound Doppler machine during three NMES-stimulation cycles. Thereafter, the measurement tool on the ultrasound Doppler machine was used to measure the peak venous velocity and the mean of the three observed PVV was used for statistical analysis. To reduce the effect of any potential measurement errors such as inaccurate Doppler angle and/or wrong Doppler probe placement [30], the mean value of three consecutive PVV registrations was calculated both at baseline (i.e., without NMES) and at ML I and ML II for each test. The PVV was assessed during the first three stimulations after ML I and ML II, respectively, was reached. The percentual increase in PVV from baseline to ML I and ML II, i.e., the primary outcome, was calculated using the formula: (Percentual increase) = (((PVV with stimulation) – (PVV at baseline))/(PVV at baseline)) × 100.

### Assessment of Comfort

The secondary outcome was the level of discomfort for the different NMES parameters tested, measured at ML I and ML II using a numeric rating scale for pain (NRS) with 0 indicating no discomfort or pain and 10 indicating the worst imaginable discomfort or pain [31].

### Test Procedure

All participants were tested by the same trained investigator. The participants were randomized to have the test carried out on the left or right leg, but if the participant had any previous injury or surgery on one leg, the other leg was used for the tests. Nine participants were tested on the right leg and six on the left leg. There were no significant correlations between the side of the leg tested and any of the outcome variables.

Initially the participants put on the NMES pants, and the investigator made sure that all electrodes had contact with the skin and 10 ml of conductor transmission gel (Chattanooga, Eco-Med Pharmaceutical Inc., Canada) was applied on the textile electrodes. Thereafter, the participant was seated down on a gurney with the hip flexed in 60° and the knee flexed in approximately 30°. A pillow was placed under the knee to keep it in the correct position. The participants were instructed to relax fully during the stimulation. The eleven tests were performed in sequence during a single session with 1 min rest between each test, and the order of the tests were randomized for each participant. The participant could at any point, for any reason and without any explanation stop the tests.

### Statistical Analysis

All data was analyzed using SPSS version 27 (IBM Corp. Released 2016. IBM SPSS Statistics for Windows, Armonk, NY: IBM Corp.) in cooperation with a statistician. All variables were checked for skewness using the Shapiro–Wilk test and approximately half of the variables were non-normally distributed. Based on that and the relatively small sample size (*n* = 15), all variables were summarized with descriptive statistics such as median, minimum–maximum, inter-quartile range (IQR) and frequency, and the Wilcoxon signed rank test was used for the inferential statistics. The significance level in all analyses was set at *p* ≤ 0.05 (two-tailed).

In order to investigate the influence of frequency and RUD-time separately, test 1–4, 10, and 11 were combined in two different ways. To examine the effect of the frequency, the mean of the tests with and without RUD for each frequency were used (tests 1 and 2, vs 3 and 4, vs 10 and 11). For the RUD-time, the mean of the three tests with the three frequencies without RUD (test 2, 4, and 11) and with RUD (test 1, 3, and 10) were used. In order to examine the influence of duty cycle and plateau time individual tests with the same other parameters were compared, test 4, 5, 6, and 7 for duty cycle, and test 4, 8, and 9 for the plateau time.

## Results

### Hemodynamics in the Femoral Vein Using Different Q-NMES Parameters

#### Current Intensity

**Fig. 3:**
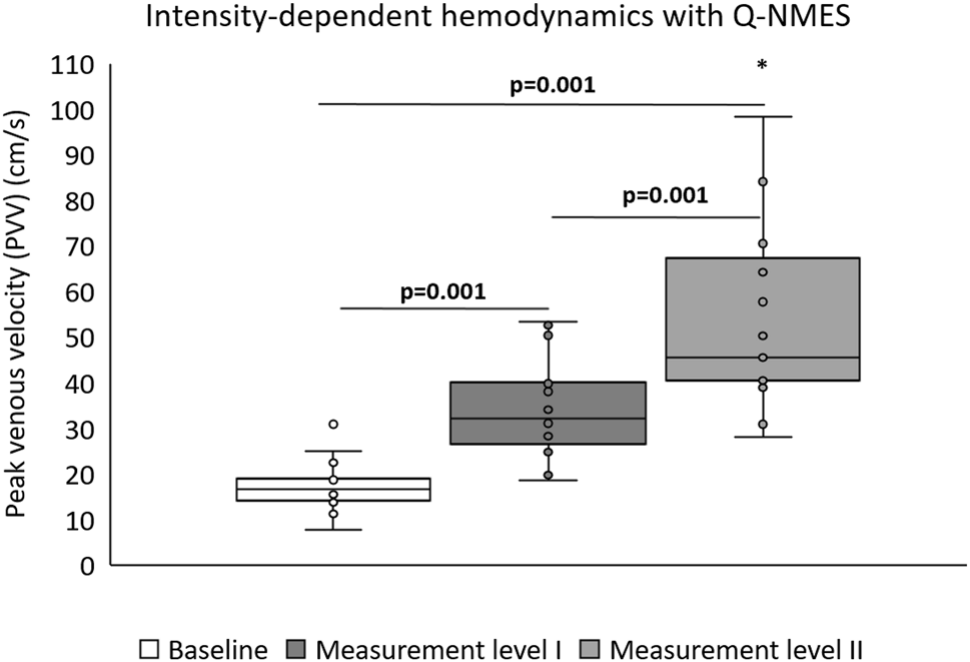
Hemodynamics in the femoral vein assessed at baseline (without NMES) and during Q-NMES at muscle contraction (ML I) and after an increase of six NMES levels from muscle contraction (ML II). The current amplitude to reach ML I was 14.1 mA (10.7–19.9 mA) and ML II was 19.6 mA (17.0–23.8 mA). *p*-values calculated with Wilcoxon signed rank test, significant values are bold. *Outlier, 143.1 cm/s. *Q-NMES* neuromuscular electrical stimulation of the quadriceps muscle, *PVV* peak venous velocity, *mA* milliampere, *ML* measurement level

The baseline median (minimum–maximum) PVV in the femoral vein among all subjects was 16.7 (11.2–30.9 cm/s). Q-NMES produced a statistically significant increase of PVV compared to baseline, at ML I with 93% (11–219%) and at ML II 173% (68–757%) (both *p* = 0.001). The PVV at ML II 45.5 (28.2–143 cm/s) was significantly higher than at ML I 32.2 (18.6–53.4 cm/s) (*p* = 0.001) (Fig. 3).

#### Frequency

**Fig. 4:**
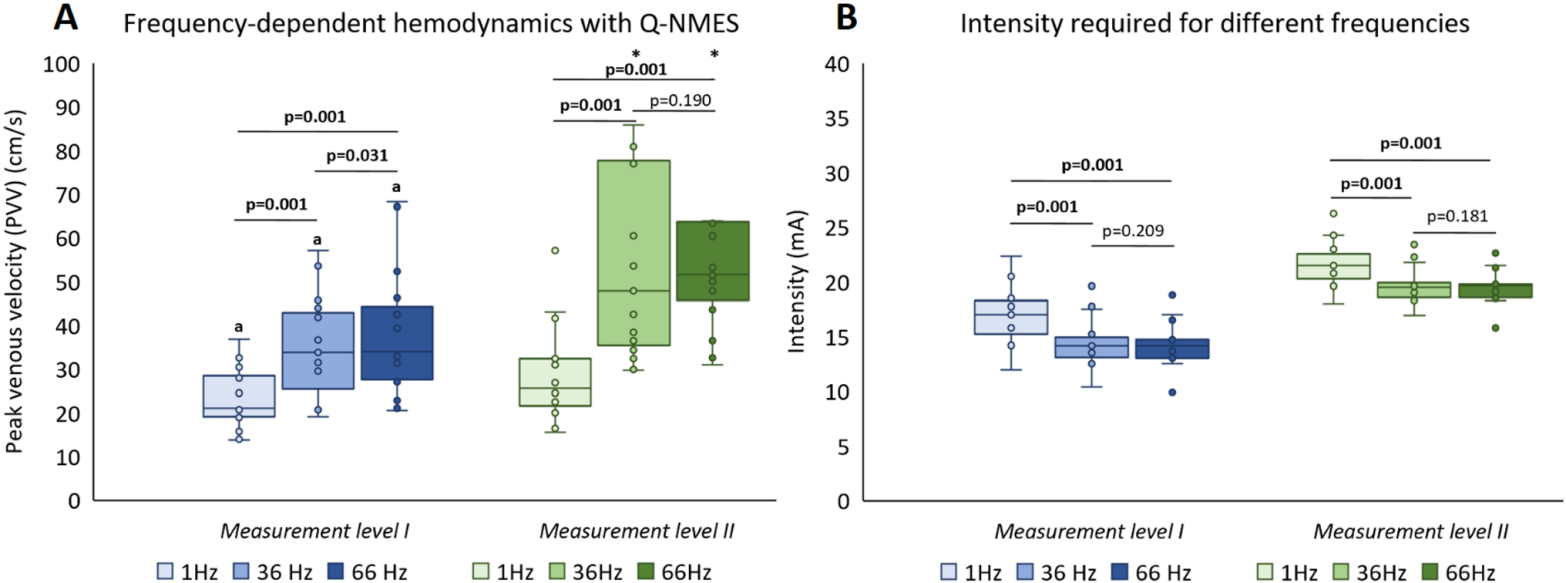
Hemodynamics in the femoral vein measured in PVV (**A**) and intensity of NMES (mA) required (**B**), at muscle contraction (ML I) and after an increase of six NMES levels from muscle contraction (ML II), for stimulation a frequency of 1 Hz, 36 Hz, and 66 Hz. Both 36 Hz and 66 Hz required significantly less intensity to reach both ML I and ML II, as compared to 1 Hz (**B**). *p*-values calculated with Wilcoxon signed rank test, significant values are bold. a: *p* < 0.01 compared to the same frequency at ML II, *outlier, 144 cm/s for ML II 36 Hz, and 146.5 cm/s for ML II 66 Hz. *Q-NMES* neuromuscular electrical stimulation of the quadriceps muscle, *PVV* peak venous velocity, *mA* milliampere, *ML* measurement level

There were significant frequency-dependent increases in PVV, at both ML I and II (Fig. 4A). Q-NMES produced statistically significant increases of PVV compared to baseline both at ML I, with 26%, 102%, and 103% (all *p* = 0.001) at 1 Hz, 36 Hz, and 66 Hz, respectively, and at ML II with 53%, 187%, and 209% (all *p* ≤ 0.002) when using 1 Hz, 36 Hz, and 66 Hz, respectively. Q-NMES at ML II resulted in significantly higher increases of PVV from baseline than at ML I for each of the frequencies tested (Fig. 4B).

#### Ramp-Up and -Down Time (RUD)

**Fig. 5:**
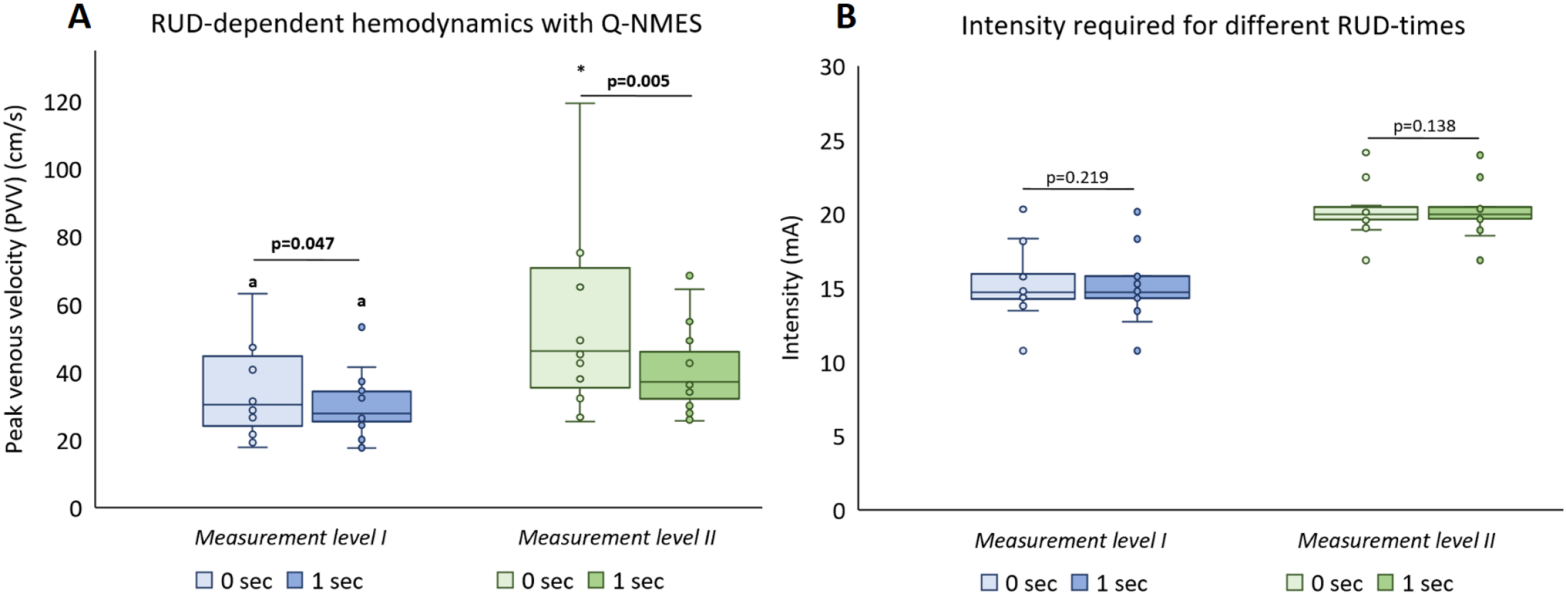
Hemodynamics in the femoral vein measured in PVV (**A**) and intensity of NMES (mA) required (**B**), at muscle contraction (ML I) and after an increase of six NMES levels from muscle contraction (ML II), for stimulation with (1 s) and without (0 s) RUD-time. *p*-values calculated with Wilcoxon signed rank test, significant values are bold. a: *p* < 0.01 compared to the same frequency at ML II, *outlier, 162.8 cm/s. *Q-NMES* neuromuscular electrical stimulation of the quadriceps muscle, *PVV* peak venous velocity, *mA* milliampere, *ML* measurement level, *RUD* ramp-up/-down time

Q-NMES produced significantly higher PVV with 0 s compared to 1 s RUD (Fig. 5A), with significant increases of PVV compared to baseline at both ML I (83% vs. 67%, *p* = 0.047) and at ML II (178% vs. 122%, *p* = 0.005). No significant differences in intensity required to reach ML I or ML II were seen with 0 s compared to 1 s RUD (Fig. 5B).

#### Duty Cycle and Plateau Time

There were no significant differences in PVV or intensity required when comparing the different duty cycles and the plateau times, at either ML I or II (Table 3).

**Table 3:** Hemodynamics in the femoral vein expressed as peak venous velocity and intensity of NMES (mA) required, at muscle contraction (ML I) and after an increase of six NMES levels from muscle contraction (ML II), for different plateau times (1.5, 4, 6 s) and duty cycles (1:1, 1:2, 1:3, 1:4)

NMES parameters	Peak venous velocity (cm/s)		Intensity (mA)	
	ML I	ML II	ML I	ML II
Plateau time (s)				
1.5	41.1 (26.2–46.8)*	54.4 (40–66.7)	13.0 (13.0–15.0)*	19.5 (18.5–20.0)
4	34.5 (25.7–48.0)*	47.1 (39.0–89.9)	14.0 (13.0–15.0)*	19.5 (18.6–20.0)
6	29.8 (26.1–42.3)*	48.3 (38.4–89.8)	14.0 (13.5–16.0)*	19.5 (19.0–21.0)
Duty cycle (on:off)				
1:1	35.7 (30.3–39.9)*	48.6 (43.3–79.8)	14.0 (13.0–15.0)*	19.5 (18.5–20.0)
1:2	34.5 (25.7–48.0)*	47.1 (39.0–89.9)	14.0 (13.0–15.0)*	19.5 (18.6–20.0)
1:3	32.0 (25.5–50.8)*	60.6 (43.3–86.7)	14.0 (13.0–15.5)*	19.5 (18.5–20.5)
1:4	37.7 (23.6–42.5)*	59.9 (48.8–88.8)	14.0 (13.0–15.0)*	19.5 (18.5–20.0)

Data are expressed as median (IQR). *p*-values are calculated with Wilcoxon sign rank test

*NMES* neuromuscular electrical stimulation, *mA* milliampere, *ML* measurement level

*p < 0.01 compared to the same parameter at ML II

### Discomfort Using Different Q-NMES Parameters

At ML I, discomfort was minimal, and the median (IQR) discomfort reported was NRS 0 (0–0), for all NMES parameters. At ML II, the discomfort level was significantly higher with NRS 0 (0–1.5), for all NMES parameters (*p* < 0.02 for all), except 1 Hz. Discomfort at 66 Hz was significantly greater than at both 1 Hz (*p* = 0.005) and 36 Hz (*p* = 0.017), and the discomfort at 36 Hz higher compared to 1 Hz (*p* = 0.016). RUD, plateau time, and duty cycle did not significantly affect the level of discomfort (Table 4).

**Table 4:** Discomfort according to NRS during NMES

NMES parameters	ML I	ML II	*p*-value, ML I vs II
Frequency (Hz)			
1	0 (0–0)	0 (0–0)	0.180
36	0 (0–0)	0.5 (0–1.0)*	**0.007**
66	0 (0–0)	1.0 (0–2.3)*^†^	**0.005**
RUD-time (s)			
0	0 (0–0)	0.7 (0–1.3)	**0.005**
1	0 (0–0)	0.7 (0–1.0)	**0.007**
Plateau time (s)			
1.5	0 (0–0)	0 (0–1.5)	**0.017**
4	0 (0–0)	1.0 (0–1.0)	**0.009**
6	0 (0–0)	1.0 (0–2.0)	**0.010**
Duty cycle (on:off)			
1:1	0 (0–0)	1.0 (0–2.0)	**0.010**
1:2	0 (0–0)	1.0 (0–1.0)	**0.009**
1:3	0 (0–0)	1.0 (0–2.0)	**0.017**
1:4	0 (0–0)	1.0 (0–2.0)	**0.011**

NRS was assessed during NMES at two levels of current amplitude, ML I was assessed at muscle contraction and ML II at an increase of six NMES levels from muscle contraction. The maximum reported discomfort in NRS at ML I was 0.5, while that of ML II was 4.0

Data are expressed as median (IQR). *p*-values are calculated with Wilcoxon signed rank test, significant values are bold

*NMES* neuromuscular electrical stimulation, *mA* milliampere, *ML* measurement level

*p < 0.05 compared to 1 Hz

^†^*p* < 0.05 compared to 36 Hz

## Discussion

In this study we showed that optimized parameters of LI-Q-NMES alone, using the NMES pants, with low levels of subject discomfort can significantly increase the PVV in the femoral vein compared to baseline. The venous increase exhibited an intensity-dependent relationship, with higher increases in PVV at ML II compared to ML I. Higher frequencies (36 or 66 Hz) and no RUD-time resulted in a higher increase in PVV as compared to a lower frequency (1 Hz) and 1 s RUD-time. 36 Hz compared to 66 Hz resulted in significantly less discomfort at higher current intensity (ML II).

The main finding of this study demonstrated that LI-Q-NMES alone, significantly increased the blood flow in the femoral vein, which to the best of our knowledge has not been shown before. The observation implies new means to improve femoral venous return using Q-NMES in order to prevent VTE in leg-immobilized persons. Our findings are corroborated by basic physiological research, showing that lower limb muscle contraction, including the quadriceps, has an important role in venous return by compression to the veins [32].

The known antithrombotic effect of calf mechanical thromboprophylaxis like NMES and IPC is created by increasing the venous blood flow [5, 8], but the exact increase in blood flow needed for DVT prevention is unknown [7]. Earlier studies on enhancement of femoral PVV demonstrated a twofold increase both with voluntary activation of the skeletal muscle pump [6, 33] and with a clinically used IPC device on the thigh [34]. Thus, our observed increases in PVV of the femoral vein by 2.8-fold using Q-NMES are higher than those seen with muscle activation and IPC, which presumably reflect clinically relevant proximal DVT-preventive effects of Q-NMES. However, the optimal PVV increase for DVT prevention is not known and warrants further studies.

Most earlier studies and most available DVT-preventive devices have focused on compression of the calf since this is the site where most DVTs arise due to lower limb immobilization [4]. However, most feared are proximal DVTs localized above the knee, which are more likely to produce fatal pulmonary embolism [4]. Therefore, increased femoral PVV is essential to “clean” the blood vessel walls above the knee. One study, in fact, examined simultaneous NMES of the calf and quadriceps and showed an increase of PVV by 1.4-fold in the femoral vein compared to baseline [10]. However, whether the increased femoral PVV was due to compression of the calf- or quadriceps muscle was unclear. Moreover, a 1.4-fold increase in femoral PVV is similar to that produced only by foot- or calf-NMES in previous studies [7, 9, 11], and the increased PVV is presumably insufficient to effectively prevent proximal DVTs. Thus, our novel finding that Q-NMES alone significantly can increase the PVV 2–3-fold in the femoral vein add an essential piece of knowledge to the existing literature.

The observation that the increase in femoral PVV produced by LI-Q-NMES could be enhanced by increasing the current intensity suggests an intensity-dependent relationship between the current used and the PVV produced. This finding is novel for Q-NMES, but in line with previous studies investigating the effect on PVV during calf stimulation, both in the popliteal [11, 35] and femoral vein [11, 36]. The intensity-dependent increase in PVV should be attributed to the known relationship between Q-NMES-intensity and the amount of muscle contraction that is obtained [15, 37].

Another main finding of this study was that the NMES parameters, mainly frequency and RUD-time, both affected how much the PVV was increased. The maximal PVV was obtained with 36 or 66 Hz at ML II, with significantly less discomfort using 36 Hz. In line with this, earlier studies have shown that higher frequencies result in a more comfortable and effective muscle contraction [14–16], which possibly could explain the higher increase in PVV. The observation that no RUD-time, i.e., a sudden increase from no stimulation to the set intensity, increased PVV to a greater extent than a gradual increase, was novel, but is supported from findings of IPC devices that uses direct, instead of gradual increases of intensity [28]. Contrary to our findings of no difference in discomfort with and without RUD, previous studies using NMES at high intensities, up to a maximum level of tolerance, have suggested that a RUD-time of at least 0.6–0.8 s is needed to improve comfort [15, 17]. The observed discrepancy is presumably explained by this study using LI-NMES, in which RUD-time may not be needed to improve comfort.

In this study, duty cycle and plateau time did not affect either PVV or discomfort, which partly is in contrast with previous studies, which suggested that these parameters influence comfort [14, 15]. No previous studies have to our knowledge investigated if these parameters would influence the PVV. A possible explanation to the observed discrepancy regarding comfort could be that the intensities used in this study was relatively low and therefore produced a low level of discomfort, while previous studies have used NMES at higher intensities [14, 15]. The findings suggest that when using LI-Q-NMES there is an opportunity to personalize the duty cycle and plateau time without impacting PVV or comfort on a clinically relevant level.

Another important aspect of this study was establishing that the quadriceps muscle could be effectively stimulated to significantly enhance venous return using NMES pants, with soft textile electrodes. This suggests new means to improve compliance to NMES treatment. The use of integrated textile electrodes in pants simplify the application, facilitate home care, and make the electrode placement no longer challenging, even for new users, with the potential to improve compliance [19–21]. A home-based protocol could reduce visits to clinical sites. This means not only improved quality of life for these patients but also a substantial reduction of healthcare and service costs.

One possible limitation with this study was that only healthy participants aged between 18 and 60 was included, and it is possible that patients, with different diseases and/or higher age [37] or obesity [38, 39], may not respond in a similar manner. In addition, the study population was quite small (*n* = 15). However, this was a first explorative study with the aim of investigating if Q-NMES could significantly influence the femoral blood flow. Further studies are needed to determine if NMES can be used as a mechanical thromboprophylaxis on the immobilized patient population. In addition, it would be interesting to examine how to combine calf- and Q-NMES in order to get optimal effects on lower limb blood flow. The ultrasound Doppler assessed via the textile pants may marginally affect the visibility, but will not affect results of venous velocity.

## Conclusion

NMES of the quadriceps muscle alone produces intensity-dependent increases of venous femoral blood flow with minimal discomfort. The superior parameters of LI-Q-NMES, delivered via textile electrodes in pants, were a frequency of 36 Hz, 0-s RUD, and intensity at ML II, with plateau and resting time personalized to minimize discomfort. Textile electrodes and the NMES pants could be candidates for future VTE-preventive devices, but further studies are needed to examine whether the increase in blood flow have a clinically relevant effect on DVT prevention.

### Supplementary Information

Below is the link to the electronic supplementary material.

Supplementary file1 (DOCX 17 kb)

## Abbreviations

BMI: Body mass index
DVT: Deep vein thrombosis
Hz: Hertz
IPC: Intermittent pneumatic compression
LI: Low intensity
mA: Milliampere
ML: Measurement level
NMES: Neuromuscular electrical stimulation
NRS: Numeric rating scale
PAS: Physical activity scale
PVV: Peak venous velocity
Q: Quadriceps
RUD: Ramp up/ramp down
VTE: Venous thromboembolism
