# Supplementary material for: Wearable Neuromuscular Electrical Stimulation on Quadriceps Muscle Can Increase Venous Flow
Source: Ann Biomed Eng. 2023 Aug 19;51(12):2873–82. doi: 10.1007/s10439-023-03349-0 (PMC10632221; doi:10.1007/s10439-023-03349-0)
Supplement: Supplementary file 1 — Supplementary file1 (DOCX 17 kb) [file 10439_2023_3349_MOESM1_ESM.docx]

**Supplementary table 1.** *Pre-testing of hemodynamics in the femoral vein with, and without NMES-pants, both at baseline and during stimulation at measurement level I (frequency 36 Hertz, 4 seconds stimulation time, 0 ramp-up and -down time, duty-cycle 1:2), expressed as peak venous velocity (PVV). The mean value of three measurements for each subject are presented for each condition.*

|  | **Baseline PVV (cm/s)** | | **PVV (cm/s) using NMES** | |
| --- | --- | --- | --- | --- |
| **Subject** | With NMES-pants Without NMES-pants | | With NMES-pants Without NMES-pants | |
| 1 | 18.9 | 18.6 | 51.1 | 49.7 |
| **2** | 14.4 | 14.8 | 22.5 | 23.4 |
| **3** | 11.9 | 12.3 | 19.1 | 21.0 |
| **4** | 22.5 | 21.9 | 39.2 | 38.1 |

NMES: Neuromuscular electrical stimulation, PVV: Peak venous velocity
